# Everything, Everywhere All At Once: Lifelong Learning and the Invisible Revolution

**DOI:** 10.1093/geroni/igaf122.2799

**Published:** 2025-12-31

**Authors:** Lyn Holley, Laura Donorfio

**Affiliations:** University of Nebraska Omaha, Omaha, Nebraska, United States; University of Connecticut, Farmington, Connecticut, United States

## Abstract

Although it is well established that social isolation is linked to depression in older adults, many take comfort in noting that older adults can utilize a myriad of technological advances to remain connected to others and life—cell phones, various apps, direct messaging, virtual visits, holograms, etc. A visit to the supermarket provides visible evidence of older adults using this technology and commercials reinforce this by featuring older adults utilizing not only this technology, but other types that help safeguard personal safety and health, do household chores, and have conversations. While these wonderful inventions seem to have increased our quality of life and are hard won by many advocacy organizations (e.g., GSA, NCOA AARP), we may have missed what perpetual, precipitous change requires of aging persons. Perpetual technological advances have been described as “the fourth Industrial Revolution.” Those who are the “oldest old” had the good fortune to age in an environment that gave them time to adapt. This fourth industrial revolution, however, is outpacing the length of time it takes to adapt, exacerbating the digital divide between generations. How can we help older adults adapt adequately to the constant daily rate of technological change? Lifelong learning may be key to help thwart the impact of perpetual, precipitous change, but other opportunities are increasingly inadequate. This paper discusses how older adults can remain and feel connected to others and life in a world of unprecedented technological change. Gerontologists are uniquely positioned to identify and deal with this threat. Shall we?

